# Protocol for the Induction of Subarachnoid Hemorrhage in Mice by Perforation of the Circle of Willis with an Endovascular Filament

**DOI:** 10.1007/s12975-014-0366-6

**Published:** 2014-08-16

**Authors:** Dominik Bühler, Kathrin Schüller, Nikolaus Plesnila

**Affiliations:** 1 Institute for Stroke and Dementia Research, University of Munich Medical Center, Max-Lebsche Platz 30, 81377 Munich, Germany; 2 Munich Cluster for Systems Neurology (Synergy), Munich, Germany

**Keywords:** Subarachnoid hemorrhage, Mouse, Model, Endovascular, Filament, Circle of Willis perforation

## Abstract

Genetically engineered mice are a valuable tool to investigate the molecular and cellular mechanisms leading to brain damage following subarachnoid hemorrhage (SAH). Therefore, several murine SAH models were developed during the last 15 years. Among those models, the perforation of the Circle of Willis by an endovascular filament or “filament model” turned out to become the most popular one, since it is believed to reproduce some of the most prominent pathophysiological features observed after human SAH. Despite the importance of the endovascular filament model for SAH research, relatively few studies were published using this technique during the past years and a number of laboratories reported problems establishing the technique. This triggered discussions about the standardization, reproducibility, and the reliability of the model. In order to improve this situation, the current paper aims to provide a comprehensive hands-on protocol of the murine endovascular filament model. The protocol proved to result in induction of SAH in mice with high intrapersonal and interpersonal reproducibility and is based on our experience with this technique for more than 10 years. By sharing our experience with this valuable model, we aim to initiate a constantly ongoing discussion process on the improvement of standards and techniques in the field of experimental SAH research.

## Background

Subarachnoid hemorrhage (SAH) is a subtype of stroke with a devastating outcome and a high socio-economic impact which equals almost that of ischemic stroke [1]. Therefore, it is surprising that SAH is by far less frequently investigated than other neuro-vascular diseases. Consequently, several important aspects of the molecular and cellular pathophysiology of SAH are not well defined, a fact which significantly impedes the development of novel therapeutic strategies [2]. One of the many reasons for this situation could be that murine models of SAH are technically demanding and have so far been difficult to standardize [3].

Since the late 1970s when Barry and colleagues published one of the first reports on the experimental induction of SAH in “small animals” [4], several techniques for SAH induction in rodents became popular. Several laboratories studied SAH in mice by directly injecting blood or blood components into the cisterna magna [5], by opening an intracisternal vein [6], by injecting blood into the basal cisterns [7], or by perforating the Circle of Willis at the skull base using an endovascular filament inserted through the external carotid artery [8–16]. Although none of these models fully recapitulate the sequels of human SAH at least for the acute phase after SAH, the endovascular Circle of Willis perforation model, or “filament model,” became one of the most popular murine SAH models. The main reason for this popularity is that it mimics the burst of a cerebral aneurysm and most of its sequels reasonably well and is therefore believed to have a superior clinical relevance as compared to all other available models [17]. Good examples for the translational potential of the filament model are reports demonstrating delayed cerebral vasospasm [8, 12–14], neurological dysfunction [9, 11–13, 15], brain edema formation [9, 11, 18–20], and a clinically relevant mortality of approximately 30 % [9, 11, 15, 19, 21].

Despite these very positive and useful aspects of the filament model, important features of the model are still not fully standardized between laboratories. This makes results difficult to compare and sometimes hard to reproduce. In contrast to other well-standardized murine models of acute brain injury, e.g., ischemic stroke, successful induction and the severity of the insult are often not monitored intraoperatively leading to heterogeneous results with large standard deviations and questionable statistical power. This makes randomized study designs difficult to perform and limits the value of this otherwise clinically highly relevant animal model [3]. Therefore, the aim of this paper is to suggest a protocol which may facilitate a better standardization of the murine endovascular filament SAH model between individual researchers and between different laboratories.

## Protocol

### Experimental Approach

Before starting any animal experiment, a proper sample size calculation should be performed and animals should be randomly assigned to experimental groups by an investigator blinded to the treatment and/or the genotype of the animals. These measures may be considered as being time consuming or distrustful on the first sight; however, it should be taken into consideration that a biased experiment is a much greater waste of time and resources. Personal bias is a normal, unintentional behavior of every motivated and dedicated scientist who wants to achieve novel results. Therefore, stringent randomization and blinding protocols should be an implicitness for every researcher keen to publish meaningful and sustainable results in high-quality journals [22].

Another important point which needs to be considered long before starting experiments on transgenic animals is the choice of proper controls. This is particularly important for studies using cerebro-vascular disease models since the cerebro-vascular anatomy is very different between mouse strains commonly used to produce transgenic animals, i.e., C57BL/6 and SV129 mice [23]. Hence, the same procedure may result in completely different results when performed on different strains of mice, and completely different results may be obtained when transgenic animals, which are in most cases a mixture of C57BL/6 and SV129 mice, are compared to a wrong wild-type mouse line. In order to avoid this potential confounder, we would recommend using appropriately backcrossed transgenic mouse lines (>10 generations) for experiments or littermates as controls for transgenic mice.

### Pre-operative Care

It is well known from studies in humans and animals that pre-operative conditions such as housing or stress may have a significant impact on brain function and on outcome after surgery [24]. Accordingly, it is highly recommended not to disrupt well-established social interactions between animals, e.g., by separating groups of mice which grew up together or by adding dominant males to well-established groups of animals, and to keep mice under the same housing conditions for at least 1 week prior to surgery.

Another potentially important confounder is access to food and water prior to surgery since even short-term fasting before surgery may significantly alter study results [25]. We recommend allowing mice full access to food and water prior to surgery. This results in sufficient hydration and relatively homogenous blood glucose levels, which are also known to have a large impact on the development of brain injury [26, 27].

### Pre-medication and Anesthesia

Animals should be brought to the surgery room only briefly before surgery, and anesthesia should be induced with no delay and with as little stress to the animal as possible. We would recommend inducing anesthesia in a small chamber, using 4 % isoflurane in 30 % oxygen until the animal loses consciousness. Animals are then weighted, preemptive post-operative analgesia is induced with carprofen (4 mg/kg s.c.), and anesthesia is maintained by intraperitoneal injection of fentanyl (0.05 mg/kg), midazolam (5 mg/kg), and medetomidine (0.5 mg/kg) as previously described [28]. Immediately thereafter, mice are intubated and mechanically ventilated (MiniVent 845, Hugo Sachs Elektronik/Harvard Apparatus) because SAH induces global cerebral ischemia for 2–3 min which results in respiratory dysfunction or even failure [8]. Intubation can be performed either oro-tracheally or by tracheotomy. For survival surgery, we perform oro-tracheal intubation as previously described [18–20, 28] and recently shown in a video publication [21]. As soon as mice are incubated and connected to the ventilator, the animal is placed on a heating pad pre-heated to 37 °C and a rectal temperature probe is inserted for monitoring and maintenance of body temperature. This is particularly important for mice because they quickly lose temperature during anesthesia [28].

The suggested anesthetics have relatively little impact on systemic blood pressure and cerebral blood flow. Specifically, the maintenance of a physiological and homogenous systemic blood pressure is important for the standardized induction of SAH since in addition to the size of the filament used for perforation of the Circle of Willis, the systemic blood pressure plays an important role for the severity of SAH. Another advantage of this anesthesia protocol is that it can be antagonized immediately after termination of surgery (see below). This allows the animals a rapid gain of consciousness, motor activity, and control of body temperature.

### Intraoperative Monitoring

The endovascular filament approach induces hemorrhage without visual control. Therefore, it is important to monitor the induction of hemorrhage in real time. Proper monitoring of SAH induction avoids post hoc exclusion of animals which had no hemorrhage and—according to our experience even more importantly—prevents pushing the filament too far and thereby causing additional mechanical brain damage at the perforation site.

**Fig. 1:**
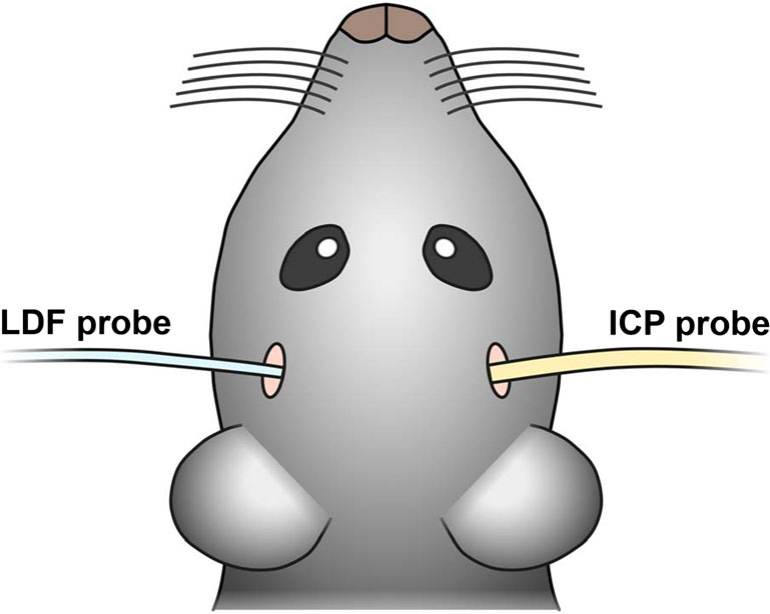
Probe positions for physiological monitoring. The laser Doppler flowmeter (*LDF*) probe is glued on the left temporal bone above the MCA territory. The intracranial pressure (*ICP*) probe is placed in the epidural space through a small borehole at the right temporal bone (adapted from Schuller et al. [21])

Monitoring the decrease of cerebral blood flow (CBF) which occurs after SAH is one possible option to monitor Circle of Willis perforation (CWp); however, we observed at different occasions that CBF may decrease without SAH [19]. This was most likely due to vasoconstriction of intracerebral vessels induced by the mechanical stimulation of the endothelium with the endovascular filament. Therefore, we suggest to monitor SAH directly by the effect of the evolving hematoma on intracranial pressure (ICP). After switching from CBF to ICP monitoring, the rate of false positively monitored SAHs dropped to zero. For this purpose, the medial part of the left temporal muscle is detached from the skull bone, a small hole is drilled into the temporal bone, and an ICP probe (ICP Express, Codman) is introduced between the bone and brain into the epidural space. The sensor is fixed with dental cement (Carboxylatzement, Speiko, Germany; Fig. 1) and ICP is recorded using a data acquisition system (PowerLab, ADInstruments). As soon as the ICP starts to rise sharply (Fig. 3a), a bleeding into the subarachnoid space takes place [21]. Upon withdrawal of the filament, the ICP rises to values close to the systemic blood pressure. Animals not showing this sharp increase in ICP or showing an increase below 50 mmHg even after a second (and final) perforation attempt should be excluded from the study. Within 5 min after the initial bleeding, values drop to around 30 mmHg. Within another 20-min observation period, ICP values stabilize around 25 mmHg (Fig. 3a). One day after the bleeding, the ICP is still elevated to 10 mmHg whereas 3 days after the hemorrhage, it normalizes at 5 mmHg [19].

**Fig. 2:**
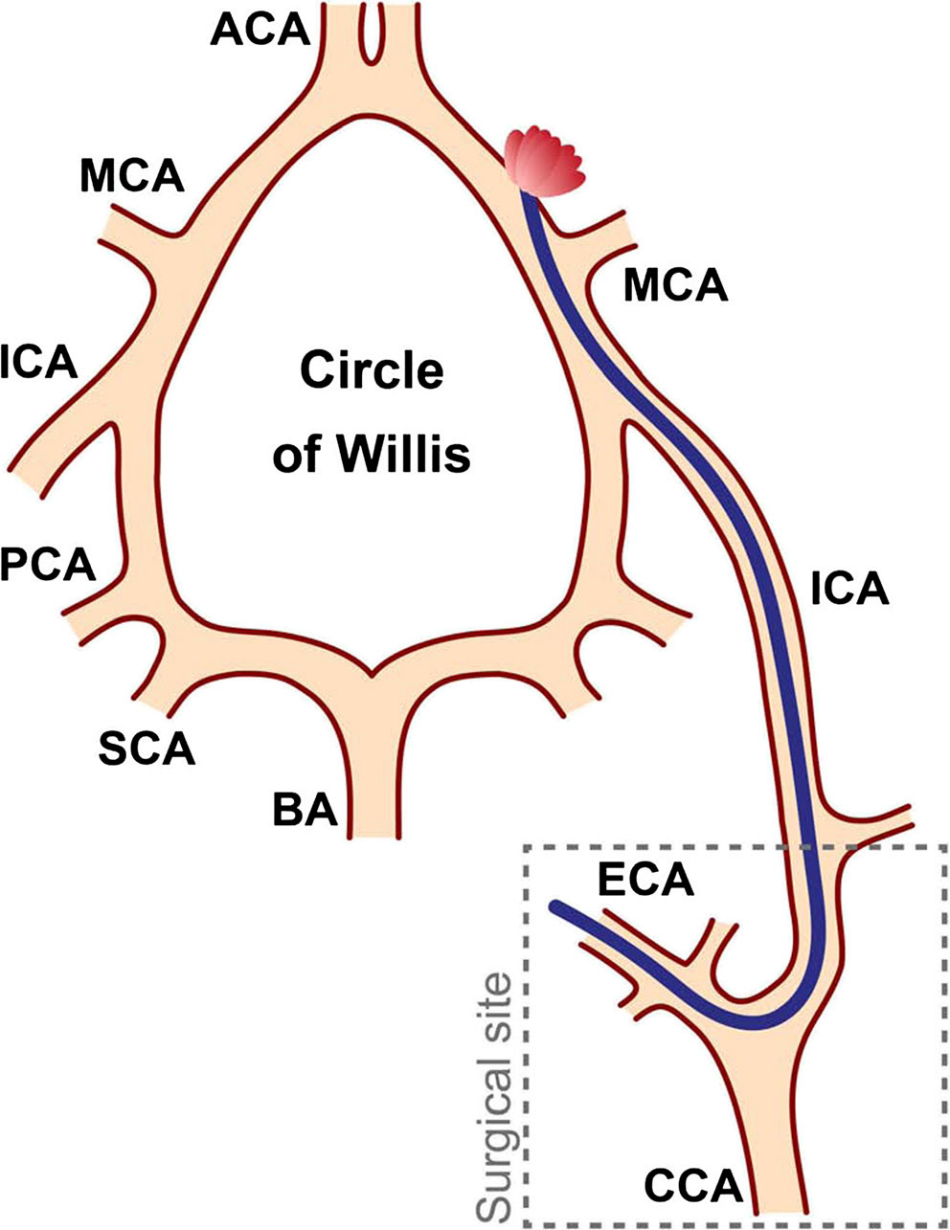
Scheme of SAH induction by perforation of the Circle of Willis. Via the surgical site at the neck, the left carotid arteries are visualized. A stiff filament (5-0 prolene, 12 mm) is introduced in the ECA, placed into the ICA and further advanced toward the Circle of Willis. By gently pushing forward, the vessel wall close to the MCA can be perforated to induce a SAH. *ACA =* anterior cerebral artery, *BA =* basilar artery, *CCA =* common carotid artery, *ECA =* external carotid artery, *ICA =* internal carotid artery, *MCA =* middle cerebral artery, *PCA =* posterior cerebral artery, *SCA =* superior cerebellar artery (adapted from Schuller et al. [21])

**Fig. 3:**
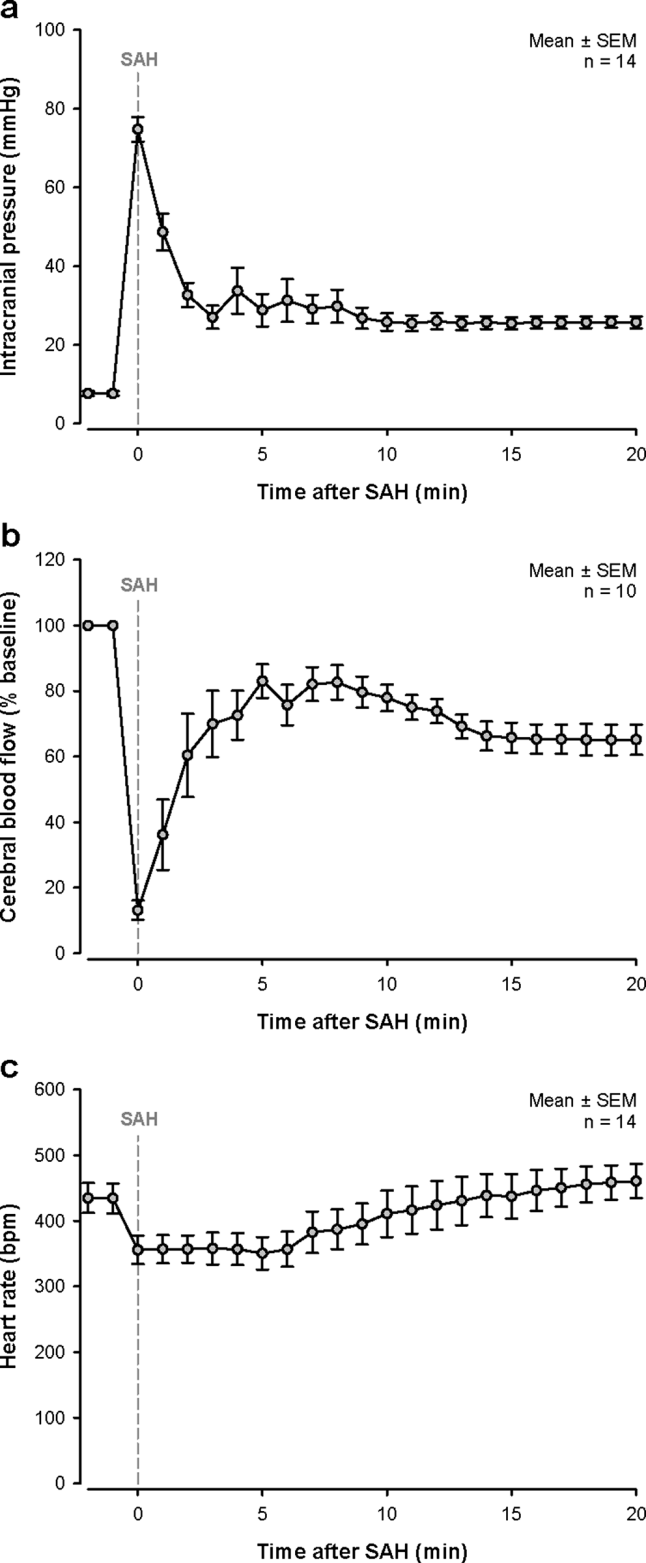
Intraoperative monitoring. **a**–**c** Continuous recording of intracranial pressure (*ICP*), cerebral blood flow, and heart rate during SAH induction (indicated by *dashed line*). SAH induction results in an immediate strong increase of ICP (**a**) which leads to a global cerebral ischemia at the same time (**b**). After a few minutes, ICP is decreasing again but stays elevated (**a**). Also, the cerebral blood flow is stabilizing but remains reduced (**b**). A drop in heart rate can be a consequence of the Cushing response to elevated intracranial pressure (**c**)

Another important parameter which determines the amount of bleeding after SAH is the systemic blood pressure [21]. The higher the blood pressure during the bleeding, the more blood is extravasating. Therefore, noninvasive blood pressure monitoring with a cuff placed around the tail of the mouse (Coda monitor, Kent Scientific) during the procedure helps to standardize the bleeding volume. The noninvasive measurement is important since this allows long-term survival of the mice after surgery without the risk of hind limb ischemia due to femoral artery catheterization. Animals with a mean arterial pressure under 60 mmHg should be excluded from the study.

Next to ICP and systemic blood pressure, also the arterial pCO_2_ needs to be measured and controlled. CO_2_ is a strong and specific dilator of cerebral vessels and therefore arterial pCO_2_ may also critically determine bleeding intensity after SAH. Arterial pCO_2_ can be reliably measured in the inspired and expired air by a microcapnometer (Capnograph 340, Hugo Sachs Elektronik/Harvard Apparatus) connected to the ventilation tube [28]. Values should be adjusted to 25–30 mmHg. This results in arterial pCO_2_ values in the physiological range (35–45 mmHg).

In order to receive additional information about regional cerebral blood flow (rCBF), a laser Doppler probe is glued on the temporal bone with cyanoacrylate glue (Fig. 1) and laser Doppler flux is measured through the intact bone. Laser Doppler recordings drop once the filament reaches the bifurcation of the MCA and reaches values close to zero when SAH occurred (Fig. 3b). As mentioned above, a drop of rCBF does not necessarily indicate vessel perforation. The heart function can be monitored by pulsoximetry on the hind paw (Mouse STAT, Kent Scientific). This noninvasive measurement provides peripheral oxygen saturation and heart rate. The high ICP after SAH induces a Cushing response, i.e., an increase in blood pressure (data not shown) and a decrease in heart rate (Fig. 3c).

### SAH Induction

**Fig. 4:**
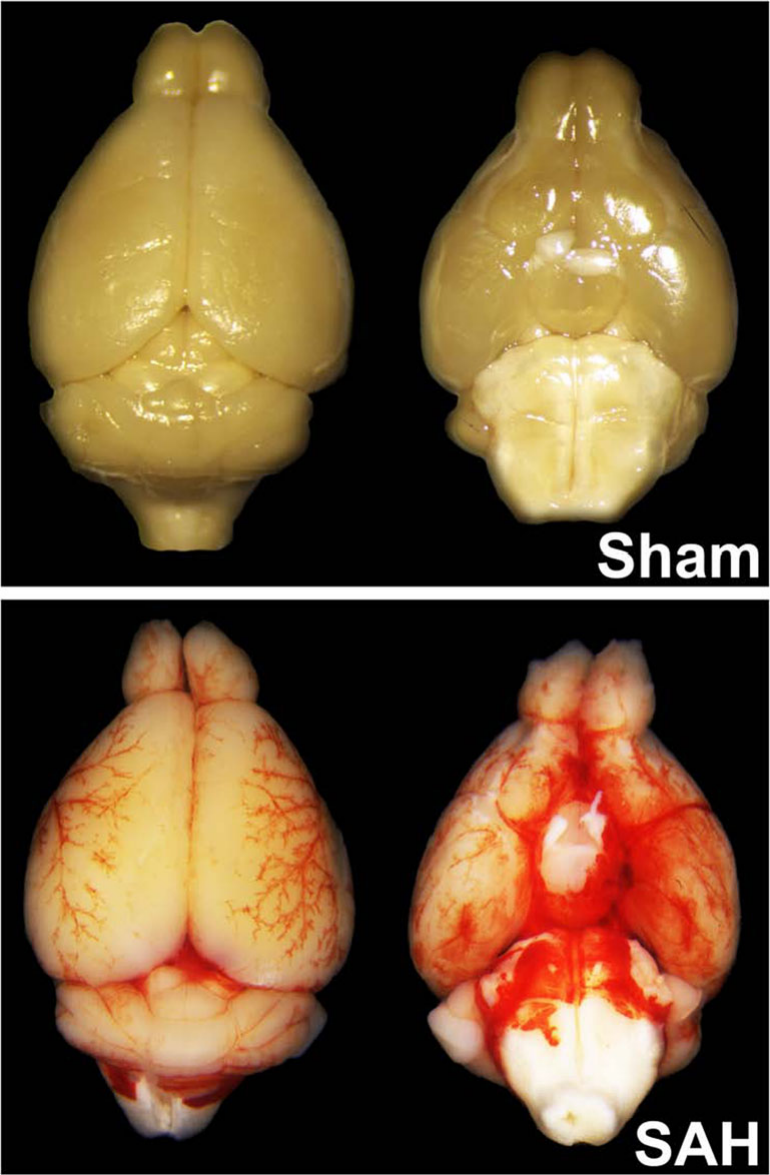
Blood distribution along arteries after SAH. Brains were removed directly after surgery to evaluate blood distribution along brain-supplying arteries. Mice were transcardially perfused with saline so that no blood remained inside the vessels. In sham-operated animals, there is no visible blood while after SAH, blood is surrounding the vessels and is distributed along the arteries (adapted from Schuller et al. [21])

First, the animal is placed in a supine position and the neck is exposed. The skin is opened in the midline. Afterward, a blunt dissection through connective tissue between the salivary glands is performed. The external, internal, and common carotid artery and their branches are exposed and partly mobilized. The external carotid artery is ligated with a silk filament and another silk filament for fixation of the perforation filament is prepared. The common and internal carotid arteries are temporarily closed with micro clips. A stiff and blunted filament (Prolene 5-0) is inserted into the external carotid artery and fixed with the pre-arranged silk filament [8, 19, 21]. After removal of the micro clips, the filament is advanced into the ICA and then further toward the brain stem (Fig. 2). A sudden increase of the ICP together with a drop of the rCBF indicates vessel perforation at the Circle of Willis (Figs. 2 and 3a, b). Once the ICP rises, the filament is withdrawn immediately from the internal carotid artery. If the ICP does not rise, the filament needs to be withdrawn completely and a second attempt to introduce the filament in to the internal carotid artery and the perforate the vessel may be performed. If this does not result in SAH, the animal needs to be excluded from the study. After SAH, the external carotid artery is ligated and the skin wound sutured. The physiologic parameters and especially the ICP are monitored for another 20 min after bleeding induction to screen for potential re-bleedings which are detected by additional sharp increased of ICP. With this technique, a preferential distribution of blood along subarachnoid vessels (Fig. 4) with little to no variation between different animals can be achieved [21].

### Post-operative Care

At the end of the surgery, anesthesia is terminated with a subcutaneous injection of atipamezol (2.5 mg/kg), flumazenil (0.5 mg/kg), and naloxon (1.2 mg/kg). In addition, 0.2 ml saline is injected subcutaneously to substitute for a possible volume loss during surgery. Mice are extubated as soon as they show motor activity. Afterward, animals are kept alone in a pre-heated incubator at 30 °C for 24 h to prevent hypothermia and are then returned together with their cage mates to their home cage. Dry and soaked food pellets together with easily accessible water are provided.

**Fig. 5:**
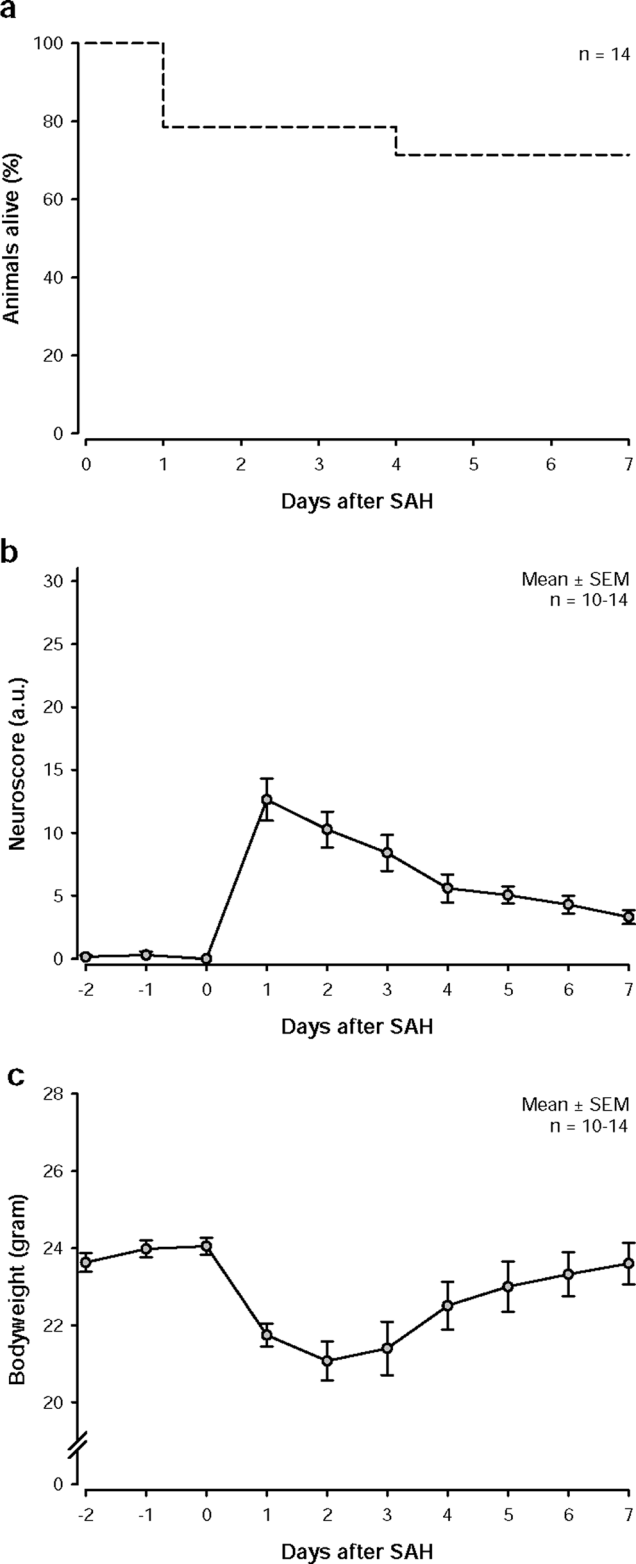
Post-operative outcome. **a** Mortality after SAH by perforation of the Circle of Willis. In this model, a mortality rate of about 30 % in the first week can be observed. **b** Daily neurological examinations. The applied neuroscore (best, 0; worst, 31; see Table 1) shows that mice after SAH induction have the biggest deficits on the first post-operative day and that they can recover gradually over time. **c** Bodyweight changes after SAH induction. Loss of bodyweight can be observed on the first 3 days after surgery. Afterward, mice start to gain weight again

Mice are observed daily for a period of 7 days. They receive daily subcutaneous injections of carprofen (4 mg/kg) and 0.2 ml saline. In our hands, this procedure results in a mortality rate of approximately 30 % mainly during the first 1–5 days after surgery (Fig. 5a). Thereafter, mice survive long term.

Neurological deficits are assessed daily for 7 days or longer using a neuroscore adjusted to the neurological deficits observed after SAH (Table 1). After SAH, mice usually score between 10 and 20 points. To receive reliable results, mice should be familiarized with the test procedure for 2 days prior to surgery. Mice not achieving the best possible performance before SAH (0 points) should be excluded from further assessment. After SAH, mice are most impaired on the first post-operative day (Fig. 5b). On the following days, mice start to recover gradually which results in lower scores.

**Table 1:** Neuroscore for SAH

Task	Criteria	Score
Consciousness	Spontaneous exploration	0
	Movements after tactile stimulus	1
	No movements (comatose)	2
Whisker movements	Present	0
	Absent	1
Hearing (turning to hand clapping)	Directed	0
	Undirected	1
	Absent	2
Motor function (per limb)	Normal	0
	Stiff	1
	Paralyzed	2
Mod. Bederson score	No obvious deficits	0
	Flexed forelimbs	1
	Lowered resistance to lateral pushing	2
	Circling if pulled by tail	3
	No spontaneous activity	5
Placing test^a^	Present	0
	Absent	1
Beam walk (3 cm, 2 cm, 1 cm)	Normal movements	0
	Improper paw placing	1
	Circling on beam	1.5
	No movements	2
	Falling off after few steps	3
	Falling off immediately	4
Total		0–31

^a^Front paws reaching ground when lifted by tail

As a sensitive indicator for general well-being, the body weight is assessed daily. The biggest loss of bodyweight can be observed on the first 3 days after surgery (Fig. 5c). Afterward, mice start to gain weight again and can almost reach their initial weight.

### Histology

**Fig. 6:**
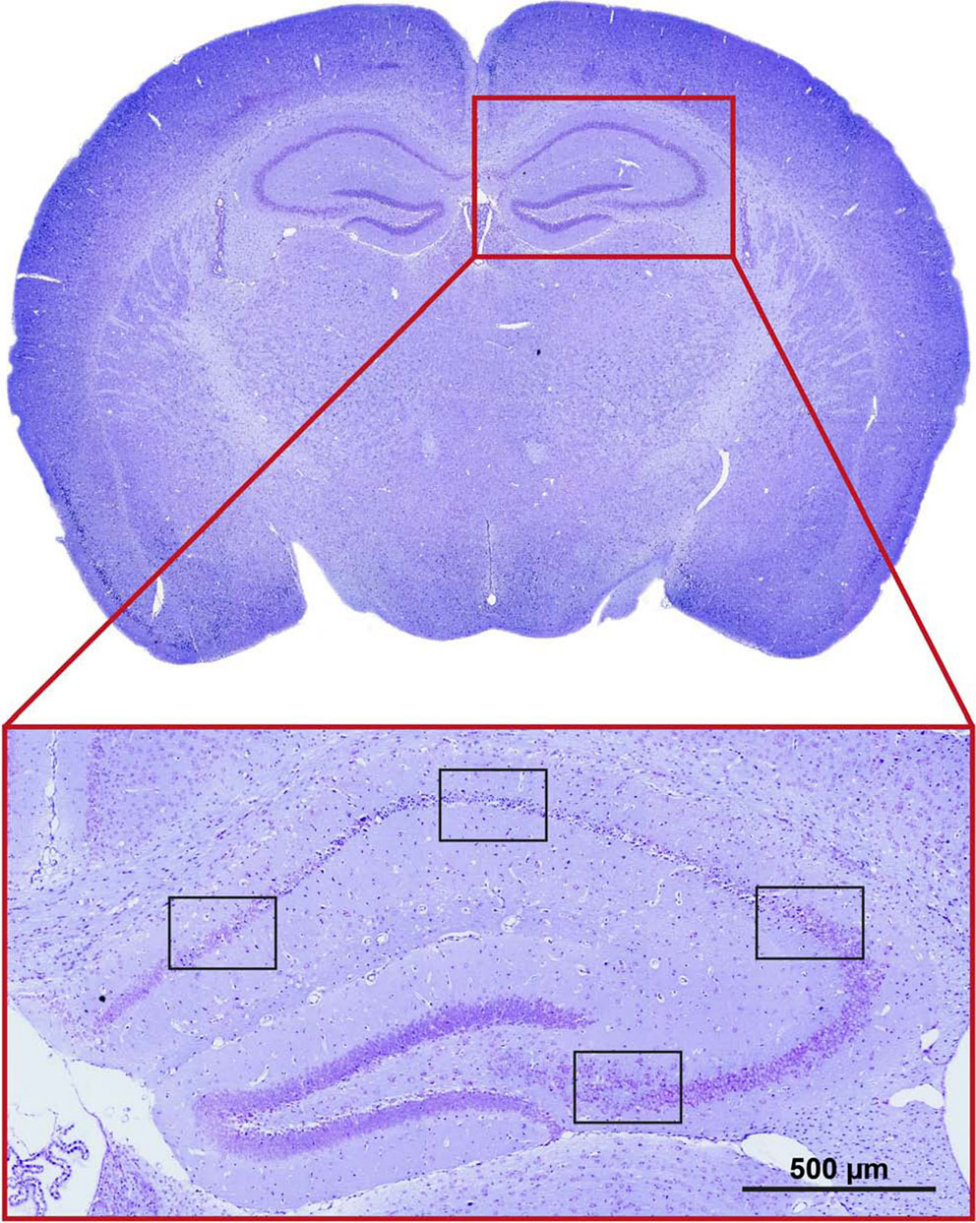
Histopathological evaluation. Neuronal damage can be evaluated, e.g., on cresyl violet stained coronal sections by counting pyramidal neurons in the CA1 region of the hippocampus

A feasible way to analyze brain damage after SAH is on formalin-fixed and paraffin-embedded brain tissue. Animals are killed by transcardial perfusion with 20 ml of saline followed by 20 ml of 4 % paraformaldehyde (PFA) in phosphate-buffered saline (PBS) at a pressure of 120 mmHg. Brains are harvested and then stored in 4 % PFA in PBS (4 °C, 24 h) for post-fixation. Afterward, brains are embedded in paraffin and 4-μm coronal sections are prepared using a microtome. On cresyl violet stained coronal sections, different regions of interest can be selected in the hippocampus to quantify viable pyramidal neurons (Fig. 6) [19].

## Summary and Conclusion

Here we present a well-established and standardized protocol for the induction of subarachnoid hemorrhage in mice by perforation of the Circle of Willis with an endovascular filament, the so-called filament model. Based on multiple publications with this protocol over the past 10 years and on repetitive validation experiments performed in our laboratory on a regular basis, the protocol proved to have a high intrapersonal and interpersonal reproducibility. Key parameters which need to be taken into consideration for reproducible induction of SAH with this method are sample calculation, randomized and blinded study design, intubation and ventilation, injection anesthesia, maintenance of physiological systemic blood pressure and arterial pCO_2_, monitoring of intracranial pressure, and a proper surgical technique. When adhering to this protocol, researchers with preexisting experience in mouse handling and surgery should be able to reproducibly induce SAH in mice after a training period of about 6 months.
